# *Notes from the Field:* Neonatal Salmonellosis Associated with Backyard Poultry — Oregon, November 2023

**DOI:** 10.15585/mmwr.mm7314a6

**Published:** 2024-04-11

**Authors:** Stephen G. Ladd-Wilson, Karen Yeargain, Samuel P. Myoda, Mansour Samadpour, Karim Morey, Paul R. Cieslak

**Affiliations:** ^1^Public Health Division, Oregon Health Authority; ^2^Crook County Health Department, Prineville, Oregon; ^3^Institute for Environmental Health Laboratories, Seattle, Washington; ^4^Oregon State Public Health Laboratory, Oregon Health Authority.

**Summary**

**What is already known about this topic?**

Salmonellosis outbreaks associated with backyard poultry involving young children have been well documented.

**What is added by this report?**

A case of backyard poultry–associated salmonellosis was identified in a newborn who was infected during the first week of life, despite living >150 miles (>241 km) from the location of the backyard flock, suggesting that even in the absence of direct exposure, backyard poultry might present a risk for salmonellosis to newborns and infants via fomites.

**What are the implications for public health practice?**

Investigation of salmonellosis outbreaks should include detailed epidemiologic inquiry regarding any potential backyard poultry exposures and follow-up environmental testing where indicated. Families with newborns and infants should be aware of the potential risks associated with owning backyard poultry.

Outbreaks of salmonellosis (infection with non-typhoidal *Salmonella*) involving young children associated with keeping backyard poultry,* including descriptions of high-risk practices such as keeping poultry inside households and kissing birds, have been well documented (*1*). During 2023 (as of October 19), backyard poultry–associated salmonellosis outbreaks were reported to CDC from 48 States and Puerto Rico; these outbreaks accounted for 1,072 cases of illness, including 247 hospitalizations (*2*). Several of these outbreaks involved multiple states and included serotypes Braenderup, Enteritidis, Indiana, Infantis, Mbandaka, and Typhimurium (*3*). During a salmonellosis outbreak investigation across multiple states (A. Lodato, CDC, unpublished data, 2023), the Oregon Health Authority, in collaboration with a local health department, investigated a case of salmonellosis in a newborn whose parents had kept backyard poultry. This activity was deemed to be routine public health surveillance by the Public Health Division of the Oregon Health Authority and did not require human subjects review.

## Investigation and Outcomes

The Oregon patient was an exclusively breastfed male newborn who was born during October 2023 at hospital A, approximately 150 miles (241 km) from the parents' home. The *Salmonella* Thompson whole genome sequencing (WGS) pattern of the isolate from the patient matched that of the unpublished outbreak strain. The newborn was discharged with his mother to a relative’s home the day after his birth. Four days later, he was readmitted to hospital B with bloody stools and lethargy, at which time a stool sample was collected for analysis and subsequently tested positive for *S.* Thompson; the WGS pattern matched the unpublished outbreak strain. Neither parent had been symptomatic, and neither had received a diagnosis of salmonellosis. The baby’s father, who tended the family’s backyard poultry approximately 150 miles (241 km) away, had been present at hospital A during the child’s birth and stayed with the child and the child’s mother at the relative’s home through the time of illness onset. The newborn had not traveled to the home where the backyard poultry were kept during the interval from his birth until his hospital admission. Twenty-seven days after this admission, nine environmental samples from the chicken bedding in the family’s backyard poultry coop (where the child’s father also had had contact) and one cloacal sample from a chicken were collected. The samples were sent to the Institute for Environmental Health Laboratories in Seattle, Washington, for *Salmonella* spp. serotyping and WGS analyses. Two of the environmental samples matched the newborn’s isolate within three single nucleotide polymorphisms^†^: clinical PNUSAS396258, and environmental CFSAN1435603 and CFSAN1435604. Samples were not collected from the parents.

## Preliminary Conclusions and Actions

The mechanism by which this newborn was exposed to this strain of *Salmonella* is not known. The newborn’s family had recently started keeping backyard poultry, having purchased the chicks in September 2023, approximately 1 month before the child’s birth. It is possible that one of the parents was asymptomatically shedding the organism and exposed the newborn during or after birth; alternatively, the organism might have been carried from the backyard farm to the newborn by fomites.

This case of neonatal salmonellosis linked to environmental isolates from a backyard poultry coop to which the newborn had not been directly exposed highlights the importance of hygiene when tending backyard poultry, especially when persons at risk for exposure are newborns and young infants whose intestinal flora and immune systems are still developing (*4*,*5*). In addition to adhering to recommended hygiene practices (*2*), families contemplating raising backyard poultry should consider the potential risk to newborns and young infants living in the household. To better understand the breadth of backyard poultry–associated salmonellosis outbreaks, state and local public health officials can conduct detailed epidemiologic inquiry around potential backyard poultry exposures, not limited to those where the patient lives, and perform follow-up environmental testing where indicated.

## Footnotes

*
https://www.cdc.gov/healthypets/pets/farm-animals/backyard-poultry.html

†
https://www.ncbi.nlm.nih.gov/
